# The dual role dilemma of liver transplantation health care professionals

**DOI:** 10.1186/s12910-023-00923-y

**Published:** 2023-07-04

**Authors:** Annette Binder, Julia Fenchel, Immanuel Lang, Anil Batra

**Affiliations:** Department of General Psychiatry and Psychotherapy, Addiction Medicine and Addiction Research Section, University Hospital Tuebingen, Tuebingen, Germany

**Keywords:** Ethical dilemma, Liver transplantation, Alcohol-related liver disease, 6-month abstinence rule

## Abstract

**Background:**

Similar to many other countries, in Germany patients with alcohol-related liver disease are obliged to prove their abstinence before being accepted on a waitlist for liver transplantation. Health care professionals (HCPs) must both treat patients and ensure that patients have proven their abstinence. The aim of this exploratory study was to develop a deeper understanding of how HCPs deal with this dual role.

**Methods:**

The study used semi-structured interviews as the source of data. 11 healthcare professionals from ten of the 22 German transplant centers were interviewed. After transcription, a qualitative content analysis was performed.

**Results:**

We found that these HCPs faced an ethical dilemma, as they must balance the roles of being both a treatment provider (the therapist role) and an assessor (the monitoring role). To solve this dilemma, the strategy seems to be a tendency for the HCPs to take on one dominant role amongst these two roles. HCPs who prefer to take on the therapist role seem to feel burdened by the 6-month abstinence rule and the obligation to monitor their patients. HCPs who prefer to take on the monitoring role tend to have negative assumptions about the patients. HCPs also reported the impression that patients perceive HCPs as more involved in monitoring and less open to the therapeutic role. From this it can be deduced that current regulations and structures lead both to stress for HCPs and to suboptimal therapy for those affected.

**Conclusions:**

The results showed that current transplantation guidelines can have a negative impact on both patient care and the burdens on the HCPs. From our point of view, there are various changes that could be made to the current clinical practice that would help solve this dilemma. For instance, integrating other assessment criteria that are more closely adapted to the health status trajectory and psychosocial background of the individual patient would be both possible and would lead to improvements in practice.

**Supplementary Information:**

The online version contains supplementary material available at 10.1186/s12910-023-00923-y.

## Introduction

Alcohol-related liver disease (ALD) is the most common indication for liver transplantation (LT) in many countries [1–4]. However, the lack of resources to enable the use of organs for liver transplant leads to exclusionary regulations and access restrictions which are perceived as unfair for certain patient groups, under which patients with ALD are subject to particularly strict requirements before they can be listed for an LT. Often, they are denied access to an LT, die before being listed or whilst waiting for an LT [5]. Although the data from European countries which supports this idea is minimal, a study from Spain showed that significantly more patients with ALD are delisted because of death or health deterioration than those delisted due to their condition improving [6]. The attitude of primary care physicians also has an impact on reduced access to listing [7].

### Legal requirements for LT in patients with ALD in Germany

In Germany, the Transplantation Act forms the legal basis for organ and tissue donation, as well as for transplantation. The German Medical Association assumes important control tasks within the German transplantation system. Guidelines for different organs were developed and are adjusted regularly by the Standing Committee on Organ Transplantation of the German Medical Association and then approved by the Federal Ministry of Health. The guidelines are legally binding for the transplant centers. The current “Guideline for Waiting List Management and Organ Placement for Liver Transplantation” of the German Medical Association requires an assessment of the compliance of potential organ recipients to ensure that there is sufficient willingness and ability to comply with and implement the medical recommendations in such patients [8]. In many countries within the European Union – including Germany – alcohol abstinence must be present for at least 6 months in patients with ALD before they can be listed for transplantation [8, 9]. Laboratory chemical detection is usually carried out by ethyl glucuronide (ETG) detection in either urine or hair. The interdisciplinary and organ-specific transplant selection committees take place within the individual transplant centers. The transplant selection committee is made up of at least five HCPs (using the ten-eyes principle) and decides on whether to admit patients to the waiting list, how to manage the waiting lists and which patients to remove from the waiting list. The composition of the transplant selection committees is determined by the corresponding organ-specific guidelines [8]. Regarding liver transplants, the HCPs from the following disciplines are entitled to vote:

- transplant surgeon
- Internist/gastroenterologist
- Anesthetist or critical care physician
- Representative of another medical discipline that is not directly involved in the transplantation process.
- Specialist in psychosomatic medicine and psychotherapy or specialist in psychiatry and psychotherapy or psychotherapist.

Depending on the clinical picture of the patient, advisory representatives of other medical disciplines such as nephrology, haemato-oncology or radiology are admitted to the transplant selection committees. In addition, representatives from nursing or from transplant coordination can participate in the transplant selection committees in an advisory capacity.

The 2015 amendment to the guideline permits an exception to be made from the six-month abstinence period provided that an external commission agrees to an inclusion on the waiting list before the end of the waiting period. Unfortunately, review criteria have not been specified for this purpose.

In Germany, to date liver transplants are performed at 22 specialized centers, each differing in their infrastructure. Therefore, it is not uniform throughout Germany to which specialist department the patients requiring an LTX are initially assigned. The cooperation of different departments (visceral surgery, internal medicine/gastroenterology, psychiatry, or psychosomatics) allows for patients to be thoroughly evaluated, a process through which their eligibility to receive an LT is assessed, while possible contraindications, such as the use of addictive substances, are also examined by specialists from the field of psychosomatic medicine or psychiatry. The laboratory-based chemical proof of abstinence that is required to enable patients with ALD to receive an LT is carried out through regular appointments at transplant centers. At these centers, appointments which enable one to receive support while maintaining abstinence can also be arranged.

### Framing and criticism of the 6-month abstinence rule

According to Primc [10], there are various interpretations that have been used to frame what the requirement of 6-months of abstinence is meant to represent. These include its use as tools for (1) diagnosis to evaluate the ability of the liver to regenerate, (2) prognosis of the transplantation success and (3) to predict drinking behavior after LT. Furthermore, as ALD is a life-threatening disease, it is essential that patients demonstrate that they are responsible enough to avoid harmful drinking behaviour in the future, thereby justifying the need for a 6-month abstinence rule. At the same time, however, the 6-month rule has been criticized for several reasons. Firstly, recent scientific findings suggest that comparably positive outcomes are achieved even with shorter abstinence periods [11]. Secondly, treatment for alcohol use disorder (AUD) may be required for improved abstinence rates, unfortunately to which not all patients may have access [12]. Thirdly, the idea that patients should be ‘responsible’ for their abstinence has been criticised as being unethical given that patients may not have the ability to remain abstinent without the right support [13, 14]. Thus, experts are calling for changes to the current regulations and demanding the inclusion of further criteria [15–17].

### Possible ethical conflicts

Health Care Professionals (HCPs) can encounter many ethical dilemmas when undertaking medical and therapeutic roles. In this article, we use the term HCPs to summarize people from various medical professions (namely physicians with different specifications and psychologists) who are directly involved in the decision-making process for LTX listing. When caring for patients with AUD who require a liver transplant (LT) due to liver cirrhosis, there are many personal challenges and potential ethical dilemmas that HCPs must face. For example, HCPs are involved in deciding whether a patient can receive a life-saving LT. In addition, they need to comply with the legal requirements and thus, their freedom to make decisions can be limited. At the same time, HCPs are responsible for their patients, being both practitioners and therapists in parallel. With donor organs being in short supply, the life-changing potential of an LT makes this decision extremely challenging. To our knowledge, little research has been done to evaluate the process underlying the listing of patients for LTs by HCPs. Thus, to date the intricacies of how HCPs make decisions during the listing process and its impact on patient care remain unknown.

### Rationale, scope and aims of the study

The qualitative interview study upon which this article is based aimed to capture the perspective of HCPs on caring for patients with ALD during the abstinence-monitoring phase before patients are listed for LT. This article will shed light on the challenges, stresses and internal conflicts of HCPs that arise from patient care during the preparation for listing.

The following questions were addressed:

1. How do health care professionals balance a caring role with a role that requires them to make controlling decisions?
2. How do HCPs feel about balancing these two roles?
3. What ethical conflicts may arise from this?
4. How do HCPs succeed in these roles when dealing with such dilemmas?

The results will help to generate a deeper understanding of the inner ethical conflicts of a HCP and how HCPs deal with them. Based on these findings, appropriate supportive measures for HCPs can be put in place, while also stimulation a reevaluation of the current regulations and practices that have been encouraged until now.

A manuscript dedicated to further research has been submitted elsewhere and addresses the following questions: (I) How are care frameworks at the LTX centers currently designed for patients with ALD who are preparing for an LTX? (II) How is proof of abstinence determined? (III) How are therapeutic interventions developed and optimised to allow the best possible care? (IV) What challenges and barriers do therapists face when complying with current care frameworks?

## Materials and methods

HCPs were interviewed by telephone from January to April 2021 about their experiences in caring for patients with ALD when preparing for an LT. The study design was approved by the Ethics Committee of the Faculty of Medicine at the University and University Hospital of Tübingen (project number: 718/2020BO2) and was performed in accordance with the Declaration of Helsinki. Informed consent was obtained from all participants.

### Reflexivity

Many identities shape the research process [18], which rather than being determined and static, involved required reflection for all the steps involved [19, 20]. Reflexivity is a process of self-consciousness and is generally considered to be crucial in qualitative research [20]. At the same time, it is considered to be of particular relevance in the context of research of ethical issues in highly professional medical contexts, as well as when working with marginalized and stigmatized groups, such as people with substance use disorders, including those with AUD.

Research can be addressed according to the relation with the (I) subject, (II) participants, and (III) research process [21]. We aspired to represent these relations as follows: (I) and (II) as covered in the following sections, and (III) as represented throughout the entire paper. In the following text, the authors reflect on the impact of the different identities that are combined in each of the authors and how this shapes the research process. As *researchers*, the focus is on the fields of addiction, substance use and health services research in Germany, with the aim of improving care and treatment for those with substance use disorders. As *psychiatric therapists*, substance use disorders are viewed as diseases, in which patients’ success in therapy is acknowledged while the reality that abstinence cannot always be achieved due to various factors is also taken into regard. As *HCPs*, we work as a part of an interdisciplinary network located at a university hospital through which collaboration with HCPs from other disciplines occurs, with each of us having different assumptions about colleagues from certain departments. These are shaped by our own positive or negative experiences in interdisciplinary cooperation.

### Interview guide

The interview guide for implementing the semi-structured interviews was developed by the research team using a multi-stage procedure [22, 23]. Based on the existing literature and the research question, relevant topics and questions were discussed and then collected by the research team. Subsequently, these were evaluated to determine which questions from an existing pool of questions were suitable for capturing the areas relevant to the research question. Subsequently, the questions were sorted thematically based on their content. In the final step, questions were assigned to different areas of the guide. Questions were assigned to the categories: “main question”, “maintenance question” or “specific follow-up question”. The guide covered the following topics:

- The structure of patient care at the LTX Center
- The needs of those affected from the point of view of the HCP
- The addiction therapies on offer at the center
- The barriers in the structure of patient care from the point of view of the HCP
- The burdens associated with patient care for those with ALD
- Suggestions and ideas for improving the supply of organs for transplantation

The interview guide can be found in Additional files (see Additional file 1).

As is usual with qualitative research approaches, the preliminary interview guideline was tested using the *field-testing* technique in order to assure intelligibility and to verify the adequacy [24]: After the first interviews, the research team discussed whether the guideline worked sufficiently for the research question or whether adjustments needed to be made. It was decided that no adjustments were necessary.

### Sampling and participants

**Fig. 1:**
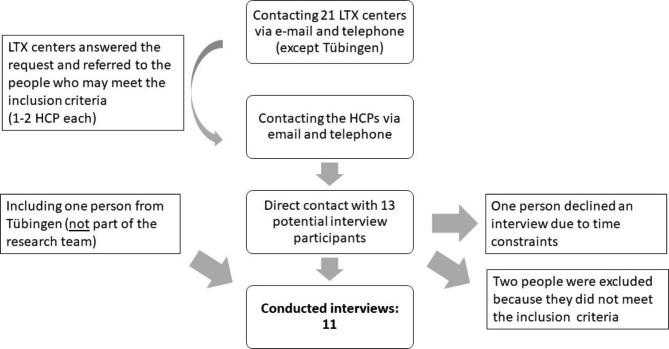
Depiction of the sampling process

In order to ensure that this study was adequately-powered, using the concept of Information Power suggested by Kristi Malterud [25], a sample size of eight people was estimated to be required. This was derived from the fact that this was an exploratory study in a homogeneous group of participants in which a high quality of dialogue was expected to be derived from the interviews with HCPs. Figure 1 provides an overview of the sampling process.

All centers offering LTs in Germany (except our own center) were contacted by email and/or telephone. HCPs responsible for the psychiatric/psychosomatic evaluation of patients with ALD and/or carrying out the EtG procedure for determining proof of abstinence before and during the listing for LTX were invited to participate in a telephone interview. All centers that responded to this query indicated that at least one or two HCPs performed the task of abstinence monitoring and/or evaluation at their center. Accordingly, attempts were made to contact these HCPs to invite them to participate in the study. To ensure homogenous sampling [26], the above-mentioned selection criteria served to obtain a sample that was as uniform as possible with regard to the professional activities. The purpose was to accurately determine differences in the responses of the interviewees. Only people who fulfilled these criteria and were willing to be interviewed were included in the sample. One person was excluded due to having had minimal contact with the relevant patient group and having had minimal involvement in both abstinence monitoring and in providing therapy for addiction prior to LTX. Another person was excluded as they had stopped working directly with this patient group some years ago and was only involved in committee and theoretical work on this topic. One person was excluded due to being unable to find the time for an interview given work-related commitments. Data saturation had occurred during the last few interviews (in interviews 9 and 10), meaning that no new subject material emerged during these final interviews. In the broadest sense, based on the concept of snowball sampling [26], another interview was conducted following these final interviews which included a staff member from our own center who was not involved in the study. It was assumed that the new information may have arisen from the organizational processes in our own center and therefore that the interview could provide valuable additional information. Structure and treatment offers related to addiction therapy from the LTX centers were one of the initial main research questions addressed. However, apart from the structural differences reported (which were not the main focus of this manuscript) this interview revealed no new content, therefore recruitment was terminated due to the aforementioned data saturation. The inclusion of one participant from our center in the sample also proved to be helpful for reasons of reflexivity, since the everyday knowledge from the research team could be applied an analytical level through targeted delineation in the form of an interview with a person not involved in the research process.

A total of 11 people from 10 centers were included in the sample. The characteristics of the participants are presented in Table 1. The guide-based interviews carried out by telephone lasted between 18 and 36 min (*M =* 26 min). The transcription was carried out by a commercial service provider.

**Table 1:** Sample - Characteristics of the interview participants

N = 11 Interviews	Average	Minimum	Maximum
Age	46 years	34 years	64 years
Work experience (general)	11 years	6 years	30 years
Work experience with LTX candidates with AUD	6,5 years	2 years	17 years
Specialist or in further training for the following specialties:	- Psychiatry and psychotherapy - Psychosomatic medicine - General surgery *In addition*: - One general doctor - One psychologist		
Leading position (senior physician, head of department, etc.)	7 participants (64%)		

### Data analysis

The analytical process was performed as a qualitative content analysis according to Kuckartz [27]. The first analysis steps, which included the creation of the category system, was performed by (AUTOR1).

Next, the discursive validation was undertaken by the entire research team, in which both the content of the material and the hierarchization of categories were evaluated, and the category system was selectively adapted accordingly. This was followed by coding, whereby text passages from the interviews were assigned to a category within the categorization system, as supported by the analysis software MAXQDA. Going through the material also served to estimate the validity of the category system. For quality assurance, the material was coded separately by two different people (AUTOR1 + AUTOR2). Thereafter, the results were discussed collectively. Furthermore, in order to increase intersubjectivity, an assignment was defined in the event of mismatched coding [28, 29]. Subsequently, type-forming cross-analysis was completed using the individual categories. Particular focus was on the ethical dilemmas that HCPs face through the dilemmas in their work. In the analytical process, type formation was used to identify different approaches in dealing with such dilemmas. It is important to mention that the formation of types was not derived from the complete interviews. Rather, sections of text, independent of belonging to a specific interview, were used for the formation of categories to which information from interview responses were assigned accordingly. This meant that assignments to different categories could be made within one interview - especially since ambivalence towards this sensitive topic within individual interviews was also visible.

### Intersubjectivity

In order to improve the quality of the overall analysis and to ensure reflexivity, perspectives obtained outside of our own research team were considered during the entire analysis process by selecting parts of the material and the category system which were then presented and discussed in the research workshops “Qualitative Methods” of the Center for Public Health and Health Services Research Core Facility for Health Services Research, University Hospital Tübingen and “Qualitative Methods in Addiction Research” of the Junior Research Group of the German Society for Addiction Research and Addiction Therapy. These insights were incorporated into the development of the research guidelines and in the creation of the category system. In all phases of the research process, the requirements of the “Standard for Reporting Qualitative Research” (SRQR) were used as a guide [30].

## Results

### Category system

The category system represents the first result of the qualitative content analysis, displaying the core topics of the interviews. In this study, the category system was primarily created inductively and contained 7 main categories. Categories 1–4 represented the structure of patient care at the LTX center and categories 5–7 represented the views about the barriers in patient care, burdens on practitioners associated with patient care and suggestions on how care and treatment for these patients could be improved. A total of 427 text passages were assigned to subcategories. The category system can be seen in more detail in Table 2.

**Table 2:** Presentation of the structure of the category system

Main	Subcategory
1. Structure of the LT Centre	1.1 Spatial structure
	1.2 Organisational structure
	1.3 Frequency and duration of addiction medical care services
2. Abstinence control	2.1 Laboratory chemical evidence of abstinence
	2.2 Proof of therapy
3.Inclusion of third-party history	3.1 Third-party history determined by relatives
	3.2 Third-party history determined by family doctors and external practitioners
4. Support services at the LT center	4.1 Psychotherapeutic and psychoeducational support at the center
	4.2 Support in arranging offers within the institution
	4.3 Support in arranging offers outside the LT center
5. Barriers to addiction treatment for the patient group	5.1 Attitudes of practitioners towards patients with addiction
	5.2 Barriers to support arising from role conflict (assessor vs. therapeutic role)
	5.3 Insufficient financing of offers that are considered useful
	5.4 Structural barriers in the addiction help network
	5.5 Physical limitations / somatic disease of the patient group
	5.6 Language barrier
	5.7 Fear of stigma
6. Burdens on practitioners	6.1 Decision on listing
	6.2 Personal role conflict
	6.3 Interdisciplinary role conflicts
	6.4 Structural burdens
7. Suggestions for improvement from the practitioners	7.1 Ideas on how to improve treatment
	7.2 Suggestions from practitioners as to how their professional work basis could be adapted to increase their confidence when deciding how to treat their patients

### Cross-analysis

In the following section, the results of the type-forming cross-analysis, in particular subcategories 5.2, 6.1 and 6.2, are presented. To provide insight into the material, we have included quotes from the interviews as examples. To maintain anonymity, we have removed all references of identity. For better readability, we have partially removed filler words or insertions (indicated by […]) without changing the content of the statement. The interviews and the sections within each interview were numbered. The first number in a bracket represents the number of the interview, the second represents the section within the interview.

### Deciding about the listing

The decision as to whether a patient can be listed seems to occupy many of the HCPs. Especially when patients cannot be listed, as illustrated by this quote:

*“… then to actually make this decision about it: This is now a patient who cannot get a liver transplant because the alcohol dependence is too strong. After all, this is almost a death sentence for some of the patients, especially when it comes to acute decompensation. They are virtually condemned not to get life-prolonging measures now. At the end of the day, it’s just a palliative situation. And that’s something I’m not used to as a psychiatrist. […]. That is something completely different, especially since these are also patients who are just fully conscious and who then look at you with hopeful eyes and say: I never want to drink again. Then to make the decision: I don’t believe you, and now you have to die, so to speak, I don’t think that’s easy.“* (7_31).

Some HCPs with a psychiatric, psychosomatic, or psychotherapeutic background (as in the above quote) assume that they are more burdened than colleagues from the somatic disciplines because of this. However, existence of the burden in the context of the decision against an LT was named by almost all interview participants. Nonetheless, HCPs from other disciplines were observed to describe less detail about stressful moments related to this topic:

*“Yes, so it already stays in the head. Especially when it is a decision against the patient”* (2_32).

### Ethical dilemma of the dual role

Regardless of the field of study or professional experience, almost all interview participants directly or indirectly addressed the dilemma associated with undertaking a dual role as a HCP:

*“What I also find difficult about the constellation we have is that we are the assessors and the therapists at the same time.“* (6_10).

The interview revealed a range of assessments that HCPs make of their own role or mission. Some HCPs make it clear that they view themselves in a dual role and want or feel obliged to fulfill both. Other HCPs distinguish or prioritize one of the two roles. For some, although emphasis was placed on their role in determining proof of abstinence, the therapeutic activity seemed to be the primary concern:

*“One difficulty I find is that, unlike other activities here … I usually have a clear therapeutic mandate, and then suddenly it becomes an order to check whether someone has the right to receive life-prolonging treatment.“* (7_25).

In particular, the association of the HCPs role with the decision as to whether an LT could take place seemed to be perceived as burdensome. Additionally, some HCPs classified their work with the LT patients as being less involved in the domain of care and treatment responsibility, and rather in the domain of patient assessment, as illustrated by this quote:

*“Yes, so it’s just less this medical task in the sense that it’s about the patient who is looking for help and wants a therapy offer, that he is made this therapy offer. That’s the typical medical thing. And in the liver transplant consultation you assume an assessor’s position”* (11_42).

### Assessor’s position vs. medical ethos (and scientific knowledge)

Certain HCPs indicated that a burden resulted from the discrepancy between the obligation imposed on them via monitoring abstinence and the intrinsic, professional ethical attitude of wanting to support their patients. The perceived injustice seems to act as an amplifier to the dilemma, since the requirements for abstinence monitoring do not apply to all indications for LT, nor to the transplantation of other organs:

*“I think one is this dilemma, that I am obliged by directive to monitor this abstinence of six months, from which one may only deviate in exceptional cases, but on the other hand I want to offer support to patients and also cannot understand the directive as it is formulated, because there are many other areas, where it is not so strictly controlled.“* (7_31).

Moreover, in addition to the professional ethos (having a desire / mission to help), some HCPs also emphasised a desire to act according to current scientific knowledge, as illustrated by this quote:

*“That’s something that makes a difference to me, because I don’t think it’s right scientifically and medically, the guideline, and I have to stick to it.“* (7_31).

This could also be interpreted as an inner strategy to substantiate one’s own attitude within the dilemma as scientific, i.e., ‘the right’ attitude.

### Monitoring and checking the proof of abstinence

Some HCPs see themselves increasingly in the domain of monitoring patients as having the need to *“control, check, quasi, a bit critically, suspiciously watch”* (4_34), because they have the impression that patients with AUD are often not honest. One of the interview participants described this process as *“tedious”* (4_34). Overall, although the therapeutic position does not seem to be completely abandoned here, the focus is on the monitoring of patients who are unsuitable for LT:

*“But this side, that we are not only the therapists, but that we are also the assessors and sometimes have to say: Then unfortunately we cannot list if there is no credible abstinence or no … Or they told us that [they were abstinent], but the hair EtG was positive”* (4_34).

Some seem to define their experience and competence based on their ability to find violations in the abstinence rule and the ability to not be influenced by the information provided by patients, as the following quotes illustrate:

*“I’ve been doing this for eleven years now. And as a result, of course, you also have a certain nose and can no longer be told so much, I have to say.“* (1_76).

*“The patients who do not comply with abstinence are fished out or found out quite well in advance.“* (5_18).

### Advocate of the patient vs. advocate of the organ

In addition to the dilemma of having to fulfill different assignments or different tasks as an HCP, there are other questions which HCPs must consider. Firstly, does the care of the HCP primarily apply to the patient who is to be listed? Secondly, to what extent do they feel responsible for the organ that is to be transplanted (successfully)? Accordingly, HCPs across disciplines indicated such dilemmas:

*“They always say so beautifully: The psychiatrists are the advocates of the patients, and the surgeons are the advocates of the liver.“* (2_30).

However, there are also ambivalences within HCPs as to what they feel responsible for during patient care, as illustrated by this quote:

*“Personally stressful… So of course, it’s a big responsibility that you have there. On the one to the donor organ, on the other hand to the recipient.”* (10_22).

### Negative assumptions and indications of structural disadvantage

In particular, HCPs who saw themselves more in the monitoring domain, and thus as ‘advocates of the liver’, tended to associate patients with AUD with negative assumptions. They described patients who tended to *“lie”* (4_34) to the practitioners and *“who also have a very strong bagatellizing share”* (4_29). Other HCPS see *“this certain dishonesty in the context of addiction”* (1_74) and classify patients as not credible:

*“In fact, our experience is that patients credibly telling you they don’t drink alcohol – that it doesn’t exist. Without objective proof, you don’t know what’s happening.“* (5_16).

People with AUD were also described as *“patients who also naturally have personality problems and are sometimes difficult”* (4_34). Some HCPs described patients with AUD as *“unpleasant patients”* (11_44) or stated that many HCPs do not enjoy working with this group of patients:

*“It’s not the case that everyone is now scrambling for this patient clientele.“* (4_38).

### Coping within the dilemma

The burden of not being able to provide all patients with the required LT seemed to concern many HCPs. Some of the HCPs described feeling a sense of relief from this concern as they believed they were obeying the formal criteria of the abstinence rule by doing so, as illustrated here:

*“Sometimes it is also the case that the patients are very seriously ill and may die if they do not get a liver soon. And that’s why there is sometimes pressure in it. And then the formal really helps us. Then the formality helps us, that we should also have objective data that prove abstinence over half a year”* (4_36).

Interestingly, for some, the presence of a 6-month abstinence rule (given from the outside) seemed to help, given that HCPs could distance themselves from the contradiction of facing their own inner demands as an HCP to provide the best possible care for a patient and the given reality of not being able to list a patient for LT. In line with this, following the requirement of a 6-month abstinence period made it more bearable for HCPs who were unable to help:

*“I think I’ll push it away a bit and say: We can’t help with every disease. So, there is the possibility, but our hands are also tied at some point - we have these guidelines.“* (3_34).

### Effect of the dual role on the doctor-patient relationship

HCPs stated that the dual role as reviewer and therapist is also perceived by patients as a special constellation (*“We have a contact that, I think, is often viewed ambivalently by patients, because you are also involved in a kind of assessing function. Even if we try to make it open and of course don’t go in there judging, etc., I think it’s still a special conversation situation for the patients.“* (8_26)), where HCPs had the impression that patients first assume their role in assessment and examination and thus have doubts about whether the HCPs will be able to provide appropriate therapeutic support despite many HCPs wanting to offer it:

*“My impression is that most patients actually perceive this more as a test: Does he believe me that I am already abstinent? Does he believe me so that I can now receive a liver transplant? So that the willingness to take help is not particularly large there. "* (7_25).

Other HCPs seem to focus less on the difficult position they can be in given the assumptions made by patients, and instead believed that it was the patient’s responsibility to contact the HCP if they were willing to receive therapy, as illustrated by this quote:

*“And whether they then use and accept the offers for themselves, they decide for themselves.“* (10_22).

## Discussion

The results of our qualitative study show that the process of evaluating and monitoring abstinence in patients with ALD is challenging for HCPs, even while attitudes and perspectives differed between HCPs irrespective of their specialty. Corroborating previous findings [31], the decision against listing is a burden for HCPs. Furthermore, there are various sources of tension that create ethical dilemmas for HCPs at work.

### Principle of care - for whom?

In this study, HCPs demonstrated a desire to care for patients and also expressed a great sense of responsibility toward doing so. However, there were differences in regard to which aspects the HCPs were more committed. Sometimes the focus was on the patient who was in the HCP’s direct care, sometimes the HCPs, owing to the lack of organs, felt more obligation to support a ‘successful’ LT and thus responsible for the potential donor organ. In 1979, Beauchamp and Childress reconstructed four principles for the biomedical field [32] which are now known as the classical principles of medical ethics apply. One of these is the principle of care (benevolence), which obliges the practitioner to act actively in order to promote and benefit the well-being (especially the life, health, and quality of life) of the patient [32]. Traditional medical ethics formulates a similar principle (Salus aegroti suprema lex), which is superior to all others [33]. In modern ethics, all four principles are on the same level [32]. In view of the scarcity of organs, criteria are discussed or applied both in the regulation of organ donation and in the access to the waiting list or for transplantation, which depend on simultaneous consideration of multiple ethical and moral principles [34]. In the study presented here, HCPs who tended to see themselves in the role of a practitioner or ‘patient advocate’ seemed to have a more supportive and benevolent attitude towards patients with alcohol dependence. They also seemed to be more convinced that patients with AUD could be successfully treated through adequate therapy and thus tried to arrange for therapy to be put in place. Supporting this idea, studies have shown that the integration of addiction treatment programmes in LT centers produces positive outcomes by improving adherence and reducing relapse [35–37]. Specialized therapies are often accepted by patients and may fulfill unmet needs for psychiatric and addictive treatment [38]. This implies that it is important that clinicians in LT centers at least indicate to patients with AUD that there are effective therapies available and that clinicians give patients hope for successful treatment. Some experts postulate that promoting openness between the patient with AUD and the transplant team will increase the chance of a successful therapeutic outcome [39]. Whether it is possible to both build a viable therapeutic relationship within the framework of the current regulations and undertake the dual role of an HCP remains open. In regard to a planned LT, however, the term ‘care’ may also apply to how the patient responds following the transplant. Accordingly, HCPs who see themselves in the role of the advocate of the organs seem to tend to classify the 6-month rule as a way of estimating whether adherence to abstinence is likely by the patient after the transplant. Historically, the rationale follows that patients who adhere to abstinence prior to an LT are better able to adhere to abstinence following an LT. While this has been suggested as a reason to justify fixing the 6-month rule, there is minimal data to support evidence of it [40]. The 6-month rule has also been associated with the assumption that ALD is ‘self-inflicted’ due to alcohol consumption. Regardless, since the 90s publications have been presenting this 6-month abstinence period is a matter of course [37][38], and, despite lacking scientific evidence for its validity, the moralizing attitude associated with this rule remains.

### Promoting a stigmatizing attitude?

HCPs who identify more with the domain of being a “liver advocate” or with ensuring abstinence seem more likely to describe AUD patients in stereotypical ways and believed that patients were responsible for their addiction. Interestingly, both surgeons and psychiatrists expressed similar thoughts. It is well known that AUD patients particularly suffer from stigma and with it often assume that they themselves are to blame for their disease [41, 42]. Schomerus et al. showed the continued persistence of the image of AUD as a “weakness of character” [41]. This is congruent with our results, which also revealed negative perceptions of the patients from the perspective of the HCPs. Other studies from a different context have shown similar results: A qualitative study of the perception of people with SUD and AUD by their caregivers shows that distrust was more common than trust. Individuals were rated as “someone who is untrustworthy and always tries to take advantage of any offer of help”. In addition, people were described as “manipulative” by health care providers [43]. The assumption that patients are lying is also common among nurses working with people with SUD, although nurses conceptualize lying as part of the condition and, in contrast, trust in patients has been identified as important by the nurses themselves [44]. People with AUD are also at risk of suffering structural stigmatization [45], which is exemplified by widespread negative attitudes of HCPs towards patients with substance use disorder and alcohol use disorder [46]. Questions about the distribution of financial resources in the health care system are often used to measure the stigmatization of people with certain diseases. For example, a study from Germany showed that medical students were most willing to decrease health care funds in the field of AUD treatment, which suggests a stigmatizing attitude of future HCPs [47]. Furthermore, HCPs have shown a negative bias when assessing the chances of success following an LT and have a tendency to describe liver disease as “self-induced” [48]. Overall, LTX in ALD is symbolic of the entire discussion about resource rationing in the case of so-called self-inflicted diseases [49]. On a theoretical level, personal responsibility for one’s own health seems to be a useful component in health systems based on justice and solidarity. At the same time, authors such as Buxy point out that social and environmental factors are not the same for all people, while concurrently health knowledge in large parts of the population is not sufficient, so that the theoretical considerations cannot be applied on a practical level [50]. Some authors hold the opinion that the issue of self-inflicted illness should not be considered when evaluating patients with alcohol use disorder for liver transplantation [16, 51, 52]. Here, the 6-month rule - referring to the framings described in the introduction - is viewed as a kind of advance payment that the patients have incurred to demonstrate their readiness for taking personal responsibility and their ability to demonstrate a high likelihood for successful abstinence henceforth. These views persist, even while this criterion has not been scientifically proven and too result in highly stigmatizing attitudes [16]. In view of the results of our study, the question arises to what extent the 6-month rule and the associated role of HCPs in ensuring its implementation promote a stigmatizing attitude in HCPs.

### Personal responsibility?

A review on “Ethical aspects of solid organ transplantation in patients with SUD” revealed two types of personal responsibility attributed to individuals [53]. On one hand, there is the responsibility related to developing an addiction and on the other hand, the responsibility to seek treatment. In this study, the second point was explicitly addressed, with HCPs emphasizing that the responsibility to seek therapeutic support when abstaining from alcohol lies with the patient. Other authors argued that the ability to seek or stay in therapy, which are influenced by factors beyond one’s control, should be considered part of the mental illness [54]. In addition, stigmatization of people with mental illnesses, including AUD, has a negative impact on the help-seeking behavior of those affected [55]. In AUD and SUD, perceived social stigma and self-stigma seems to play a role in people’s willingness to seek treatment [56]. Accordingly, this should be considered before assuming that a patient is responsible. It is also worth noting that the dual nature of a HCP’s role (therapist and assessor) may limit the treatment options that are accessible to a patient. Therefore, frameworks that enable patients with AUD to accept therapy offers at a low threshold should be created.

### Coping within the dilemma

In our study, HCPs showed different strategies to deal with the dilemma. It seems that preferring one of the two roles (monitoring or caring) helped the HCPs to partially avoid the dilemma. Another strategy for dealing with the dilemma seems to be by emotionally distancing oneself from the patient or his/her fate by strictly focusing on the rules. With this latter strategy, HCPs could refrain from reflecting on their own ideas and attitudes, removing any personal association they had with the dilemma and thus the subsequent negative feelings associated with it. HCPs could indeed use both strategies to reduce negative feelings. Hierarchies and other barriers could make it difficult or impossible to follow one’s values in patient care or to verbalize moral conflicts, ultimately leading to dissatisfaction among HCPs [57]. It would therefore be helpful to create opportunities to address this in a protected setting through supervision or collegial intervision (also called collegial advice: a method for processing concerns from a professional context with others at a peer level without an external specialist), potentially reducing the distressing feelings of negative affect for HCPs. As studies involving transplant hepatologists [58] and surgeons [59] show that negative affect is a predictor of burnout, providing such opportunities may be beneficial in reducing negative outcomes such as burnout in HCPs. Adapting these interventions based on therapeutic insights, addiction medicine and individual patient trajectories will help maximise their potential.

### Strengths and weaknesses

The chosen method was well suited to gain insights into the experiences of HCPs. Due to the homogeneity of the sample and the high quality of the dialogues, the sample size used here allowed the study to be well-powered [25]. A limitation could be that people who disagree with the current regulations may have been more likely to sign up to participate, perhaps with the aim of expressing their dissatisfaction or because they hoped to contribute to a structural improvement by participating in the study. Additionally, interviewing HCPs with different levels of professional training may have contributed to a greater variance in interview responses in regard to the attitude towards and the assessment of this dilemma. Nonetheless, this variation maps the inhomogeneous treatment landscape in Germany during the monitoring of the 6-month abstinence rule and the provision of therapy. It should also be noted that different attitudes towards and assessments of the dilemmas were found within different disciplines.

### Conclusion & implications

In the care and evaluation of patients with ALD prior to LTX, HCPs operate in a context characterized by partially contouring legal frameworks and medical/therapeutic requirements. In particular, the legal framework is controversial from both a medical and ethical perspective. The impact of these frameworks on the professional actions of HCPs, and thus indirectly on patient care, has not yet been investigated. To fill this research gap, we examined the ethical dilemmas that arise and how HCPs deal with them. In addition to a burden of deciding whether or not a patient could be listed for LTX, other burdens were found in the dilemma of fulfilling the dual roles of treatment provider and assessor. In resolving this dilemma, HCPs seem to tend to feel more committed to one of the two roles. HCPs who prefer the therapist role seem to feel burdened by the 6-month abstinence rule and the obligation to monitor their patients. In doing so, these HCPs feel they cannot fulfill their paired role as therapist and meet the needs of patients with AUD. HCPs who prefer to assume the monitoring role tend to have negative assumptions about patients. This may involve adopting a stigmatizing attitude towards the group of patients with AUD. HCPs also feel that patients perceive them as more involved in monitoring and less open to the therapeutic side of the role. This could lead to a decrease of help-seeking behaviour among these patients and, consequently, a worsening of their chances of maintaining abstinence.

In summary, we found that current transplant guidelines may have a negative impact on both patient care and the burden on healthcare professionals. In our view, several changes could be made to current clinical practice that would help resolve this dilemma. For example, it would be conceivable to integrate other assessment criteria that are more adapted to the disease course and psychosocial background of the individual patient. In addition, supportive measures in dealing with the stress and reflecting on one’s own professional attitude would be desirable for HCPs. In our study, weaknesses of the current legal framework and a need for change regarding the current guideline and its clinical implementation were pointed out. In future research, concrete suggestions for improvement would have to be collected. This could be realized by interviewing the same people about this issue or by conducting focus group discussions with a panel of experts, representatives of the commission of the German Medical Association and affected persons. In addition, further research is needed to capture patients’ perspectives on both the addiction treatment process and on the abstinence monitoring prior to LTX in order to better understand the impact of the HCP’s dual role on patients. Participatory research approaches that involve both patients and their families could be helpful in finding solutions that meet their needs.

## Electronic supplementary material

Below is the link to the electronic supplementary material.

Additional File 1: Interview guide

## Data Availability

The datasets generated and analysed during the current study are not publicly available due to the individual privacy of the interviewed participants but are available from the corresponding author on reasonable request.

## Abbreviations

AUD: Alcohol use disorder
SUD: Substance use disorder
ALD: Alcohol-related liver disease
HCPs: Health care professionals
LT: Liver transplantation

## References

[1] Cholankeril G, Ahmed A (2018). Alcoholic liver disease replaces hepatitis C virus infection as the leading indication for liver transplantation in the United States. Clin Gastroenterol hepatology: official Clin Pract J Am Gastroenterological Association.

[2] Müller PC, Kabacam G, Vibert E, Germani G, Petrowsky H (2020). Current status of liver transplantation in Europe. Int J Surg.

[3] Goldberg D, Ditah IC, Saeian K, Lalehzari M, Aronsohn A, Gorospe EC (2017). Changes in the prevalence of hepatitis C virus infection, nonalcoholic steatohepatitis, and alcoholic liver disease among patients with cirrhosis or liver failure on the waitlist for liver transplantation. Gastroenterology.

[4] Hughes CB, Humar A (2021). Liver transplantation: current and future. Abdom Radiol.

[5] Sarkar M, Watt KD, Terrault N, Berenguer M (2015). Outcomes in liver transplantation: does sex matter?. J Hepatol.

[6] Pose E, Torrents A, Reverter E, Perez-Campuzano V, Campos-Varela I, Avitabile E (2021). A notable proportion of liver transplant candidates with alcohol-related cirrhosis can be delisted because of clinical improvement. J Hepatol.

[7] Perut V, Conti F, Scatton O, Soubrane O, Calmus Y, Vidal-Trecan G (2009). Might physicians be restricting access to liver transplantation for patients with alcoholic liver disease?. J Hepatol.

[8] Bundesärztekammer. Richtlinie gemäß § 16 Abs. 1 S. 1 Nrn. 2 u. 5 TPG für die Wartelistenführung und Organvermittlung zur Lebertransplantation. Deutsches Ärzteblatt. 2019.

[9] Mathurin P, Lucey MR (2020). Liver transplantation in patients with alcohol-related liver disease: current status and future directions. Lancet Gastroenterol Hepatol.

[10] Primc N (2020). Das „framing “der sechsmonatigen Karenzregel in der Lebertransplantation. Ein Beispiel für sprachlich vermittelte Deutungsmuster zur Eingrenzung des Indikationsgebietes. Ethik in der Medizin.

[11] Herrick-Reynolds KM, Punchhi G, Greenberg RS, Strauss AT, Boyarsky BJ, Weeks-Groh SR (2021). Evaluation of early vs Standard Liver Transplant for Alcohol-Associated Liver Disease. JAMA Surg.

[12] Carrique L, Quance J, Tan A, Abbey S, Sales I, Lilly L (2021). Results of early transplantation for alcohol-related cirrhosis: integrated addiction treatment with low rate of relapse. Gastroenterology.

[13] Martens W (2001). Do alcoholic liver transplantation candidates merit lower medical priority than non-alcoholic candidates?. Transpl Int.

[14] Zambrano A (2016). Why alcoholics ought to compete equally for liver transplants. Bioethics.

[15] Mellinger JL, Volk ML (2018). Transplantation for alcohol-related liver disease: is it fair?. Alcohol Alcohol.

[16] Batra A, Wiesing U. Zur ethischen und wissenschaft-lichen Fragwürdigkeit der „Karenzklausel “bei alkoholabhängigen Patienten auf der Warteliste zur Lebertransplantation. Hogrefe AG; 2018.

[17] Bramstedt KA, Jabbour N (2006). When alcohol abstinence criteria create ethical dilemmas for the liver transplant team. J Med Ethics.

[18] Olukotun O, Mkandawire E, Antilla J, Alfaifa F, Weitzel J, Scheer V et al. An analysis of reflections on researcher positionality. Qualitative Rep. 2021;26(5).

[19] Palaganas EC, Sanchez MC, Molintas VP, Caricativo RD. Reflexivity in qualitative research: a journey of learning. Qualitative Rep. 2017;22(2).

[20] Malterud K (2001). Qualitative research: standards, challenges, and guidelines. The lancet.

[21] Savin-Baden M, Howell-Major C. Qualititative research: The essential guide to theory and practice. Qualitative Research: The Essential Guide to Theory and Practice Routledge. 2013.

[22] Helfferich C. Leitfaden-und Experteninterviews. Handbuch Methoden der empirischen Sozialforschung. Springer; 2019. pp. 669–86.

[23] Kruse J (2015). Qualitative Interviewforschung.

[24] Kallio H, Pietilä AM, Johnson M, Kangasniemi M (2016). Systematic methodological review: developing a framework for a qualitative semi-structured interview guide. J Adv Nurs.

[25] Malterud K, Siersma VD, Guassora AD (2016). Sample size in qualitative interview studies: guided by information power. Qual Health Res.

[26] Patton MQ. Qualitative research & evaluation methods: integrating theory and practice. Sage publications; 2014.

[27] Kuckartz U (2018). Qualitative inhaltsanalyse. Methoden, Praxis, Computerunterstützung.

[28] Helfferich C. Die Qualität qualitativer Daten. Springer; 2011.

[29] Kruse J, Schmieder C. Qualitative interviewforschung: Beltz Juventa; 2014.

[30] O’Brien BC, Harris IB, Beckman TJ, Reed DA, Cook DA (2014). Standards for reporting qualitative research: a synthesis of recommendations. Acad Med.

[31] Volk ML, Biggins SW, Huang MA, Argo CK, Fontana RJ, Anspach RR (2011). Decision making in liver transplant selection committees: a multicenter study. Ann Intern Med.

[32] Beauchamp T, James F (1979). Childress Pririnciples of Biomedical Ethics.

[33] Vollmann J, Urban, Wiesing, Herausgeber (2004). Ethik in der Medizin. Ein Studienbuch.

[34] Lauerer M, Kaiser K, Nagel E (2016). Organ transplantation in the face of donor shortage-ethical implications with a focus on liver allocation. Visc Med.

[35] Donnadieu-Rigole H, Jaubert L, Ursic‐Bedoya J, Hanslik B, Mura T, Gamon L (2019). Integration of an addiction team in a liver transplantation center. Liver Transpl.

[36] Erim Y, Beckmann M, Tagay S, Beckebaum S, Gerken G, Broelsch CE (2006). Stabilisation of abstinence by means of psychoeducation for patients with alcoholic liver disease awaiting liver transplantation. Z Psychosomat Med Psychother.

[37] Ness C, Hardie K, Holbeck M, Saucedo-Crespo H, Auvenshine C, Steers J et al. Integration of addiction treatment and behavioral therapies in Comprehensive Liver Transplantation Care to augment adherence and reduce Alcohol Relapse. J Liver Transplantation. 2021:100061.

[38] Erim Y, Böttcher M, Schieber K, Lindner M, Klein C, Paul A (2016). Feasibility and acceptability of an alcohol addiction therapy integrated in a transplant center for patients awaiting liver transplantation. Alcohol Alcohol.

[39] Weinrieb RM, Van Horn DH, McLellan AT, Lucey MR (2000). Interpreting the significance of drinking by alcohol-dependent liver transplant patients: fostering candor is the key to recovery. Liver Transpl.

[40] Marroni CA, Fleck AM, Fernandes SA, Galant LH, Mucenic M, de Mattos Meine MH (2018). Liver transplantation and alcoholic liver disease: history, controversies, and considerations. World J Gastroenterol.

[41] Schomerus G, Matschinger H, Angermeyer MC (2014). Attitudes towards alcohol dependence and affected individuals: persistence of negative stereotypes and illness beliefs between 1990 and 2011. Eur Addict Res.

[42] Kilian C, Manthey J, Carr S, Hanschmidt F, Rehm J, Speerforck S (2021). Stigmatization of people with alcohol use disorders: an updated systematic review of population studies. Alcoholism: Clin Experimental Res.

[43] Lago RR, Peter E, Bógus CM (2017). Harm reduction and tensions in trust and distrust in a mental health service: a qualitative approach. Subst Abuse Treat Prev Policy.

[44] Johansson L, Wiklund-Gustin L (2016). The multifaceted vigilance–nurses’ experiences of caring encounters with patients suffering from substance use disorder. Scand J Caring Sci.

[45] Schomerus G, Matschinger H, Angermeyer MC (2006). Preferences of the public regarding cutbacks in expenditure for patient care. Soc Psychiatry Psychiatr Epidemiol.

[46] Van Boekel LC, Brouwers EP, Van Weeghel J, Garretsen HF (2013). Stigma among health professionals towards patients with substance use disorders and its consequences for healthcare delivery: systematic review. Drug Alcohol Depend.

[47] Hoffmann H, Koschinowski J, Bischof G, Schomerus G, Rumpf H-J. Medical Students’ Readiness for Cutbacks in Health Care Expenditures of Alcohol-Dependent Individuals. Sucht. 2020.

[48] Van J, Aloman C, Reau N (2021). Potential Bias and Misconceptions in Liver Transplantation for Alcohol-and obesity-related liver disease. Am J Gastroenterol.

[49] Sharkey K, Gillam L (2010). Should patients with self-inflicted illness receive lower priority in access to healthcare resources? Mapping out the debate. J Med Ethics.

[50] Buyx AM (2008). Personal responsibility for health as a rationing criterion: why we don’t like it and why maybe we should. J Med Ethics.

[51] Ursic-Bedoya J, Faure S, Donnadieu-Rigole H, Pageaux G-P (2015). Liver transplantation for alcoholic liver disease: lessons learned and unresolved issues. World J Gastroenterology: WJG.

[52] Beresford T (2001). The limits of philosophy in liver transplantation. Transpl Int.

[53] Notini L, Vasileva D, Orchanian-Cheff A, Buchman DZ (2019). Ethical issues associated with solid organ transplantation and substance use: a scoping review. Monash Bioeth Rev.

[54] Bailey D, Pathak S, Ahmad N (2013). Is liver transplant for alcohol-related end-stage liver disease appropriate?. Br J Hosp Med (2005).

[55] Clement S, Schauman O, Graham T, Maggioni F, Evans-Lacko S, Bezborodovs N (2015). What is the impact of mental health-related stigma on help-seeking? A systematic review of quantitative and qualitative studies. Psychol Med.

[56] Hammarlund R, Crapanzano K, Luce L, Mulligan L, Ward K (2018). Review of the effects of self-stigma and perceived social stigma on the treatment-seeking decisions of individuals with drug-and alcohol-use disorders. Subst abuse rehabilitation.

[57] Friedrichs A, Kraus L, Berner M, Schippers G, Broekman T, Rist F (2013). Adaption einer niederländischen Zuweisungsleitlinie für Patienten nach qualifiziertem Alkoholentzug–Ergebnisse einer Delphi-Befragung. Suchttherapie.

[58] Pourmand K, Schiano TD, Motwani Y, Kriss M, Keefer L, Patel A. Burnout among transplant hepatologists in the United States. Liver Transpl. 2021. 10.1002/lt.26375 34826182

[59] Jesse MT, Abouljoud M, Eshelman A (2015). Determinants of burnout among transplant surgeons: a national survey in the United States. Am J Transplant.

