# Supplementary material for: The dual role dilemma of liver transplantation health care professionals
Source: BMC Med Ethics. 2023 Jul 4;24:46. doi: 10.1186/s12910-023-00923-y (PMC10318701; doi:10.1186/s12910-023-00923-y)
Supplement: Supplementary file 1 — Additional File 1: Interview guide [file 12910_2023_923_MOESM1_ESM.docx]

| **Main questions** | **Maintenance questions** | **Specific follow-up questions** |
| --- | --- | --- |
| Introductory questions |  |  |
| **Please briefly outline how the care of patients awaiting listing for LTX or transplantation who need to demonstrate abstinence is managed at your facility.** | How exactly does this work at your facility? How often? What is the content of the therapy sessions? |  |
| Care needs of affected persons/their relatives | | |
| **How do you assess the care situation of these patients?** | Is the current treatment/support service appropriate?  What are the barriers/problems in care? | How does that work for you at the facility? |
| **In what areas do they need your help?** | What exactly do those affected need? |  |
| **Do you have any suggestions on how to improve care for those affected?** | Do they have any suggestions on how to meet care needs? | Do they have any other concrete ideas? |
| **How do you assess the role of relatives in the care of those affected?** | Do you have any examples of this from your everyday life? |  |
| **How does your personal burden of caring for this group of patients differ from the burden of other physician responsibilities?** | How do these burdens manifest themselves?  Are there cases/decisions that you still think about for a long time? | Can you give me an example of that? |
| Closing question |  |  |
| **Is there anything else important from your side that we haven't talked about yet?** |  |  |
